# Disparities in Beef Tapeworm Identification Rates in the Abattoirs of Gauteng Province, South Africa: A Descriptive Epidemiologic Study

**DOI:** 10.1371/journal.pone.0151725

**Published:** 2016-03-23

**Authors:** Daniel Nenene Qekwana, James Wabwire Oguttu, Dries Venter, Agricola Odoi

**Affiliations:** 1 Section Veterinary Public Health, Department of Paraclinical Sciences, Faculty of Veterinary Sciences, University of Pretoria, Pretoria, South Africa; 2 Department of Agriculture and Animal Health, College of Agriculture and Environmental Sciences, University of South Africa, Johannesburg, South Africa; 3 Gauteng Department of Agriculture and Rural Development, Veterinary Services Directory, Pretoria, South Africa; 4 Biomedical and Diagnostic Sciences, College of Veterinary Medicine, University of Tennessee, Knoxville, Tennessee, United States of America; Charles University in Prague, CZECH REPUBLIC

## Abstract

**Background:**

Bovine *Taenia saginata* cysticercus infections (also called bovine cysticercosis or beef measles) is usually diagnosed in cattle only during post-mortem meat inspection. The aim of this study was to investigate the identification rates of these infections in and to identify predictors/determinants of variations in the identification rates in abattoirs in Gauteng province, South Africa.

**Methods:**

Retrospective data for over 1.4 million cattle carcasses inspected in 26 abattoirs between January 2010 and December 2013 were used for the study. The identification rates (proportion of bovine *Taenia saginata* cysticercus positive carcasses) were computed and generalized estimating equations used to identify predictors/determinants of identification rates.

**Results:**

The overall identification rate was 0.70% (95% CI: 0.45, 0.95). Significantly (p< 0.05) lower rates were reported during summer (0.55%) than other seasons. Some geographic areas reported significantly (p<0.05) higher rates than others. The identification rates in high throughput abattoirs was significantly (p<0.05) higher (RR: 9.4; 95% CI: 4.7–19.1) than in low throughput abattoirs. Similarly, the identification rates among animals from feedlots were significantly (p<0.05) higher (RR: 1.6; 95% CI: 1.7–3.5) than those from non-feedlot sources. No significant (p>0.05) association was identified between identification rates and either the number of meat inspectors per abattoir or the provider of inspection services.

**Conclusion:**

Although no significant association was found between identification rates and provider of inspection services, follow-up studies will need to be done to specifically investigate the potential conflict of interest arising from the fact that abattoir owners hire meat inspection services directly. Capture of abattoir surveillance data needs to include farm address and for each case to be reported separately. Finally, information on the type of identified cysts (alive or calcified) need to be collected to help better estimate risk to consumers. This study provides useful baseline data to guide future studies, surveillance and control efforts.

## Introduction

Cattle are the intermediate hosts for the larval stages of *T*. *saginata*, while humans act as definitive hosts. Thus, for the life cycle of *T*. *saginata* to be complete, there must be a link between humans and animals [1]. Therefore, humans acquire *T*. *saginata* infestation when they consume raw or undercooked beef having *T*. *saginata* cysts. Grazing contaminated pastures and drinking water contaminated with the intermediate stages of the parasite passed out by humans are risk factors for *Taenia* infestation in cattle. [1, 2].

Following ingestion of *T*. *saginata* eggs by cattle, the larvae develop into cysticerci primarily in the skeletal and cardiac muscles. These cysts are fully developed in 4–5 months and measure approximately 0.5–1.0 cm. Cysts may vary in appearance depending on the degree of inflammation, necrosis, and mineralisation of the resulting lesion [3]. *Taenia saginata* cysts are potentially infective to humans by 10 weeks [1].

Infested cattle do not usually show symptoms and therefore detection of infection usually only occurs during meat inspection [4–6]. Although meat inspection has 100% specificity [7], it has low sensitivity (11.5–15.6%) [8, 9], which increases the risk of potential exposure/infection to consumers due to false negative meat inspection results.

In South Africa, all bovine carcasses have to be individually inspected for the presences of bovine *T*. *saginata* cysts by visual examination and incision of the masseter, heart, diaphragmatic and triceps muscles. Palpation of the tongue and visual examination of the offal is also prescribed. If a carcass presents a generalised infestation, the carcass and offal have to be declared unfit for human consumption and condemned. However, if the infestation is localised, the carcass could be partially passed on condition that it is treated by freezing at temperatures not exceeding −10°C for more than 14 days [10, 11]. The implication of this, is serious financial losses that occurs due to either carcass condemnation or costs of cold treatment and downgrading of affected carcasses [2, 12, 13].

According to Section 11 of the South African Meat Safety Act 40 of 2000 [14], abattoir owners are required to procure meat inspection services for their abattoirs. Meat inspection services can be provided by either companies or private individuals. Moreover, the Act stipulates that the inspection services have to be independent of the abattoir management. However, because abattoir owners pay the meat inspection service providers directly, a conflict of interest is likely to arise especially where private individuals are involved, which could adversely affect the identification rates of the condition in different abattoirs. This is a problem that needs to be addressed to enhance the meat inspection services in the country. In November of 2012, a proposal by a meat inspection working group was tabled to address the issue [10]. However, the question that arises is whether indeed the provider of the meat inspection services does influence the rate of identification of bovine *T*. *saginata* cysticercus positive carcasses in abattoirs.

The burden of bovine cysticercosis globally differs by region with high prevalence proporations reported in developing countries. Studies done in Europe have demonstrated a prevalence of 3.09% of bovine cysticercus in Belgium [15] and 1.23% in France [16]. In Africa, a prevalence of 19.7% has been reported in Ethiopia [17] and 0.2% in the North West province of South Africa [18]. However, there is no evidence of any studies that have been done to assess the prevalence of the condition among animals slaughtered in abattoirs in Gauteng province, South Africa.

In light of this, the objectives of this study were to estimate identification rates of bovine *T*. *saginata* cysticercus positive beef cattle slaughtered in abattoirs located in Gauteng Province of South Africa between 2010 and 2013 and to identify predictors/determinants of the variations in identification rates.

## Materials and Methods

### Ethics approval

This study was approved by the Animal Ethics Committee of the University of Pretoria.

### Study area

This study was conducted in Gauteng Province which is South Africa’s most populous province with an estimated population of 12.3 million people. The province is approximately 18,178 km^2^ and consists of three metropolitan municipalities (City of Johannesburg, City of Tshwane and Ekurhuleni) and two district municipalities (Sedibeng and West Rand). The two district municipalities are further subdivided into seven local municipalities for administrative purposes [19]. The province as a whole has a subtropical climate, but Johannesburg tends to be cooler than Pretoria. Gauteng Province is located in the Highveld region with an annual summer rainfall of approximately 700 mm, with December and January being the wettest months of the year. The province experiences average annual maximum temperatures of about 22°C in the south and about 25°C in the north.

### Data source

Retrospective data used in the study were from 26 abattoirs located in Gauteng Province of South Africa and covered the time period January 2010—December 2013. Approval to use the data was obtained from the Gauteng Department of Agriculture and Rural Department (GDARD) that also provided the data to the investigators. The Meat Safety Act 40 of the year 2000 requires that all animals slaughtered in South African abattoirs to be inspected and for details of the animals and findings of meat inspection to be recorded [14]. The Act defines a bovine *T*. *saginata* cysticercus positive carcass as one whose head, active muscles and red offal is found to have one or more parasitic intermediate stages of the parasite that is either alive or calcified. The information collected during meat inspection is sent to the Gauteng Department of Agriculture and Rural Development (GDARD) where it is entered into an electronic database as part of disease monitoring and surveillance.

The data used in the present study included monthly abattoir reports of the total number of cattle slaughtered, number of carcasses inspected, as well as the number of bovine *T*. *saginata* cysticercus positive carcasses identified. The study included data from both high and low throughput abattoirs located in Gauteng and registered with GDARD. A low throughput abattoir is defined as one that handles a maximum of 20 units per day [14]. However, if only one species of animals is slaughtered per day, then the maximum accepted limits are: (a) no more than 20 units if handling cattle or horses or sausage pigs larger than 90kgs; (b) no more than 40 units if handling sheep or goats, or (c) no more than 30 units if handling pigs. High throughput abattoirs, on the other hand, are classified at the discretion of the provincial executive officer based on the capacity of the lairages, hourly throughput potential relative to available equipment and facilities, such as hanging space and chiller capacity, as well as handling of rough offal [10].

### Data collection and management

The data were collated into monthly numbers of carcasses inspected at each abattoir, and bovine *T*. *saginata* cysticercus positive carcasses identified during meat inspection. The data were evaluated for missing values and any inconsistencies such as implausible values. The following variables were included in the final dataset: number of animals slaughtered and inspected, number of cysticercus positive carcasses, municipality where the abattoir was located, month of the year when animals were slaughtered and sources of the animals (feedlot or non-feedlot). For purposes of this study, a feedlot was defined as an intensive animal farming system where beef cattle are fattened prior to slaughter. Non-feedlot sources, on the other hand, included all sources of animals that did not fit the above definition.

### Data analysis

Descriptive analysis was performed to determine the proportions of bovine *T*. *saginata* cysticercus positive carcasses (presented as identification rates) by month, year, season and municipality. To assess whether the data were normally distributed or not, Shapiro-Wilks test was used. Exact Wilcoxon test was used to compare the differences in identifications rates when the data were not normally distributed otherwise Chi-square analysis was used. Statistical significance was assessed at p≤0.05.

To investigate associations between identification rates and the suspected predictors/determinants, a Generalized Estimating Equation (GEE) model was fit to the data. The dependent variable was specified as the number of bovine *T*. *saginata* cysticercus positive carcasses identified per abattoir per month, and the natural log of the number of carcasses inspected per abattoir per month was used as the offset. The error distribution and link function were specified as Poisson and log, respectively. Using this modelling framework, simple/two-way associations were first investigated to identify variables that had significant simple associations with the outcome. The following variables were assessed for the simple associations with the dependent variable: class of the abattoir (high vs low throughput), source of the animals (feedlot vs non-feedlot), meat inspection service provider (private individuals vs company), and municipality. Variables that had significant (p<0.05) simple associations with the outcome were offered for assessment in the multivariable GEE model. A backward elimination procedure with a critical p-value of 0.05 was used to identify significant predictors/determinants. All possible two-way interactions of variables in the final main effects model were assessed for significance. Since GEE is not a likelihood‐based method, Quasi-likelihood under the Independence Model Criterion (QIC), which is analogous to the Akaike Information Criterion (AIC), was used to assess model fit and identify the best correlation structure for the data.

## Results

### Descriptive analysis results

**Fig 1:**
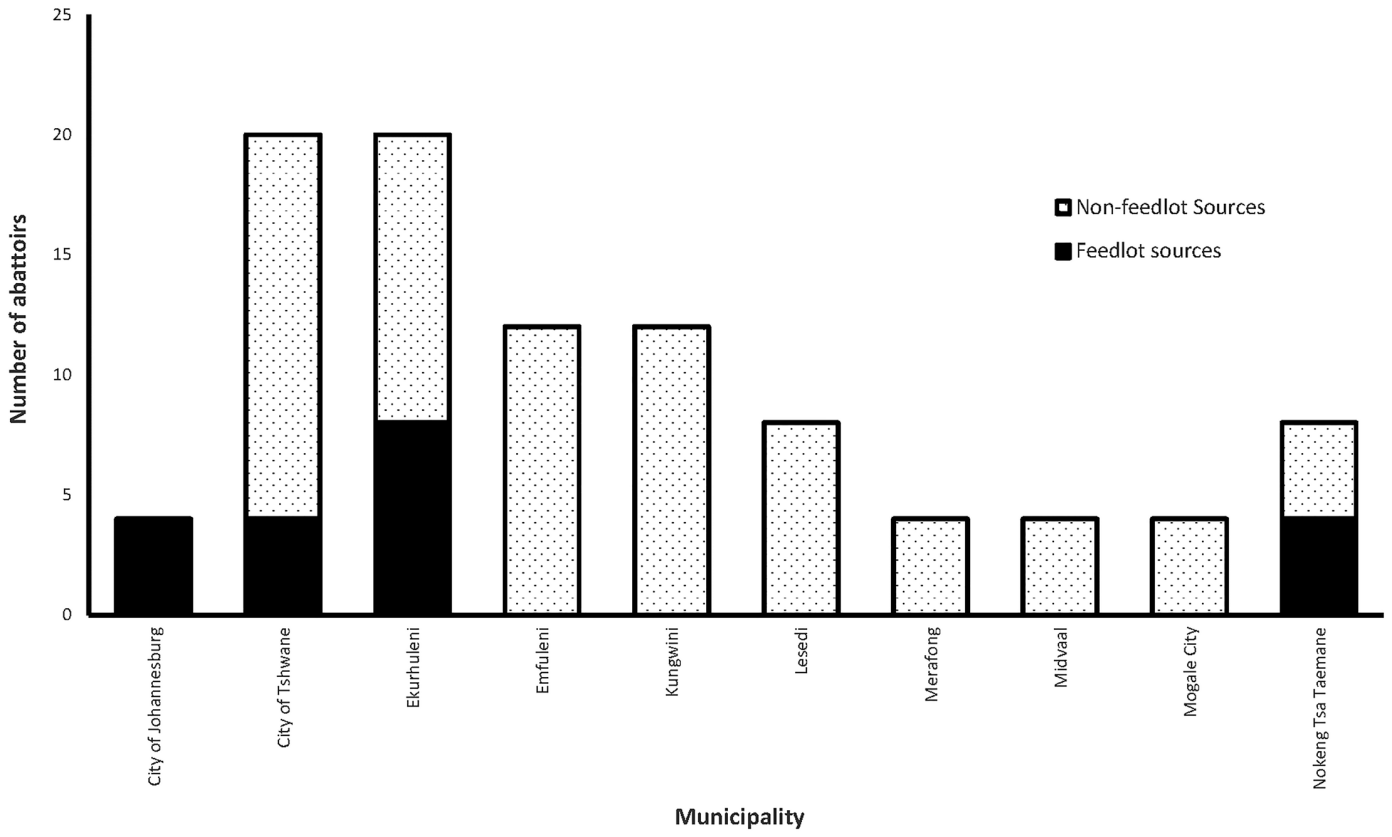
Distribution of sources of slaughtered animals by municipalities in Gauteng Province (South Africa), 2010–2013.

A total of 1,415,005 animals were slaughtered and the same number of carcasses inspected during the study period (January 2010-December 2013). Most (1,373,229) of the carcasses inspected at the abattoirs were of animals from non-feedlot sources (Fig 1). A total of 9,920 out of …. (0.70%, 95% confidence interval [95% CI]: 0.45, 0.95) cattle slaughtered during the study period were positive for bovine cysticercosis.

### Simple/univariable associations

**Fig 2:**
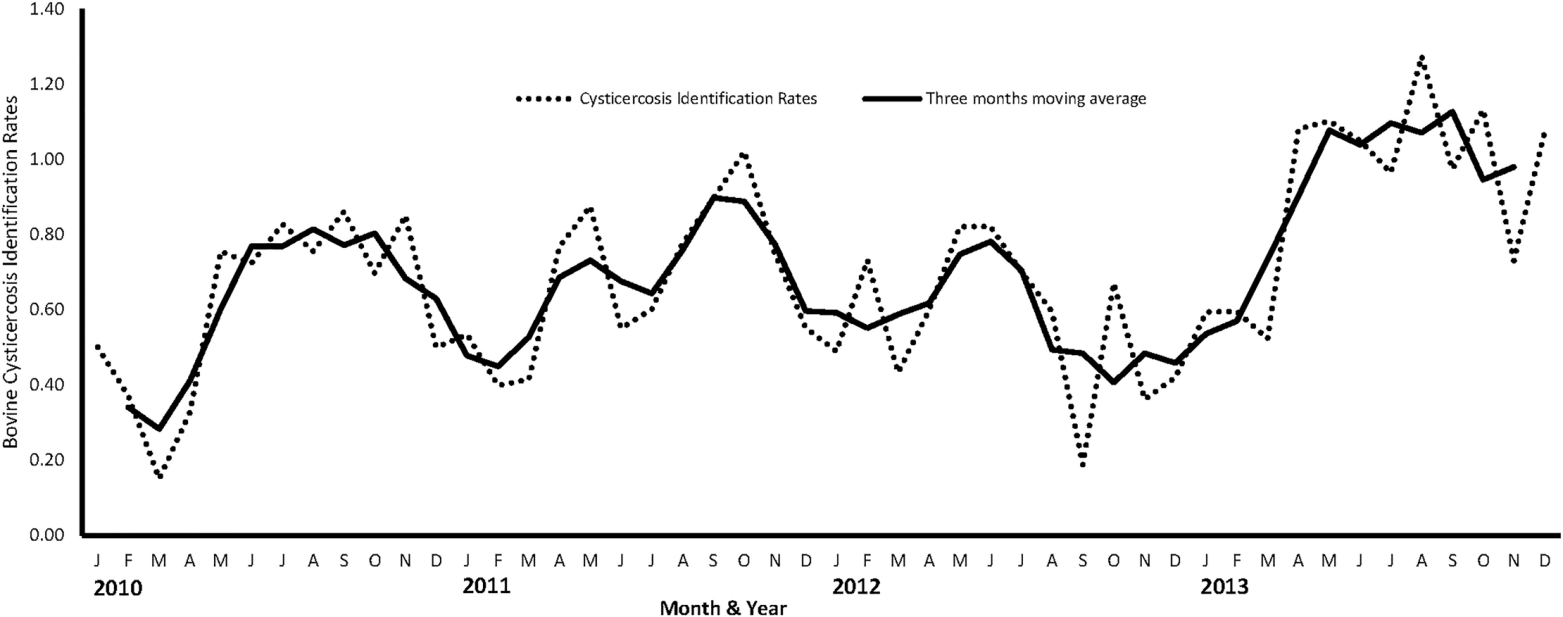
Temporal trends in monthly bovine *T*. *saginata* cysticercus identification rates in abattoirs across Gauteng Province (South Africa) between 2010 and 2013.

**Fig 3:**
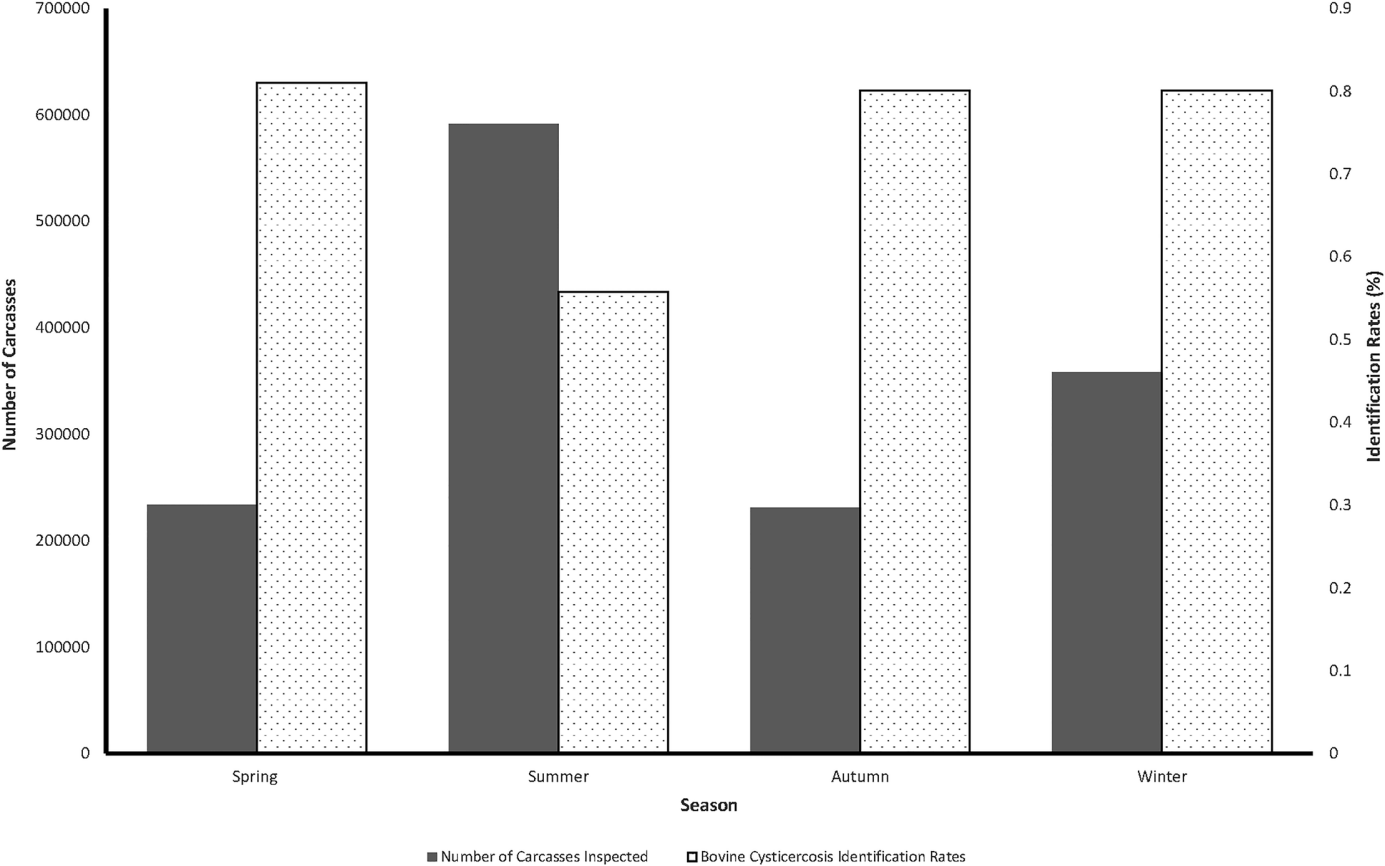
Seasonal patterns in number of carcasses inspected and bovine *T*. *saginata* cysticercus identification rates in Gauteng Province (South Africa), 2010–2015.

There were significant (p<0.05) differences in bovine cysticercosis identification rates across years, with the highest identification rates reported in 2013 and the lowest in 2012 (Table 1). There were monthly fluctuations in identification rates over the study period with summer months (November to March) having relatively low identification rates compared to the other months (Fig 2). Similar patterns were revealed by the results of seasonal analysis that also showed significantly (p<0.05) lower bovine cysticercosis identification rates during the summer months (0.55%) than other seasons. This coincided with the time when relatively more animals were slaughtered (Fig 3). Although there seemed to be evidence of an increasing temporal trend (R² = 0.46) in identification rates of bovine cysticercus positive carcasses over the study period, the observed increase was not statistically significant (p = 0.325).

**Table 1:** The total number of cattle slaughtered in Gauteng Province (South Africa) between 2010 and 2013 and proportion of bovine *T*. *saginata* cysticercus positive carcasses identified, treated and condemned.

Year	Number of abattoirs	Number of carcasses inspected	Number of bovine cysticercosis positive carcasses	Percentage (95% CI*) of bovine cysticercosis positive carcasses	Number of treated carcasses	Percentage (95% CI*) of treated carcasses	Number of condemned carcasses	Percentage (95% CI*) of condemned carcasses
**2010**	25	356,006	2, 169	0.61 (0.58, 0.63)	2, 118	0.59 (0.57, 0.62)	51	2.34 (1.78, 3.1)
**2011**	25	349,458	2, 389	0.68 (0.66, 0.71)	2, 380	0.68 (0.65, 0.71)	9	0.38 (0.13, 0.62)
**2012**	25	348,309	1, 980	0.57 (0.54, 0.59)	1, 975	0.57 (0.54, 0.59)	5	0.25 (0.03, 0.47)
**2013**	26	361,232	3, 382	0.92 (0.90, 0.97)	3, 374	0.93 (0.90,0.97)	8	0.24 (0.07, 0.40)

* CI: Confidence interval

The bovine cysticercosis identification rates were significantly (p<0.05) higher during the months of May (0.89%), October (0.88%), August (0.85%), and lower during February (0.53%) and March (0.37%) (Table 2).

**Table 2:** Monthly identification rates of bovine *T*. *saginata* cysticercus in Gauteng Province (South Africa), 2010–2013.

Month	Number of carcasses inspected	Number of Bovine cysticercosis Positive carcasses	Percentage (95% CI*) of Bovine cysticercosis Positive carcasses
**January**	100,581	535	0.53 (0.49, 0.58)
**February**	102,738	546	0.53 (0.49, 0.58)
**March**	117,741	440	0.37 (0.34, 0.41)
**April**	111,914	785	0.70 (0.65, 0.75)
**May**	119,218	1, 067	0.89 (0.90, 1.02)
**June**	116,059	902	0.78 (0.73, 0.83)
**July**	122,039	948	0.78 (0.73, 0.83)
**August**	120,025	1, 020	0.85 (0.80, 0.90)
**September**	112,709	825	0.73 (0.68, 0.78)
**October**	121,230	1, 071	0.88 (0.83, 0.94)
**November**	125,759	847	0.67 (0.63, 0.72)
**December**	144,992	934	0.64 (0.60, 0.69)

*CI: 95% Confidence interval

A comparison of the identification rates across municipalities showed significantly (p<0.05) higher rates in Nokeng Tsa Taemane (1.23%) and Mogale City (1.04%) than the rest of the provinces, while the lowest rates were reported in the City of Tshwane (0.01%). No cases were observed in the Midvaal municipality (0%) (Table 3). Identification rates for *T*. *saginata* cysticercus infection were significantly (p<0.002) higher in the high throughput abattoirs (0.024%) compared to low throughput abattoirs (0.05%). There was no significant (p<0.4226) difference between the identification rates observed among animals sourced from feedlots (0.054%) as compared to those from non-feedlot sources (0.033%). Moreover, significantly (p = 0.0295) higher identification rates were observed in abattoirs where meat inspection was performed by independent service providers (0.05%) compared to those in which the owners (0.0%) were responsible for performing meat inspection (Table 4).

**Table 3:** Bovine *T*. *saginata* cysticercus identification rates by municipalities of Gauteng Province (South Africa), 2010–2013.

Metropolitan Municipality	District municipality	Local municipality	Number carcasses inspected	Number of Bovine cysticercosis Positive carcasses	Percentage (95% CI^†^) of Bovine cysticercosis Positive carcasses
City of Johannesburg			707	4	0.57 (0.15, 1.44)
City of Tshwane			31,455	4	0.01 (0.003, 0.03)
Ekurhuleni			54,002	65	0.12 (0.09, 0.15)
	Sedibeng	Emfuleni	110,014	552	0.50 (0.46, 0.54)
		Lesedi	256,868	1,726	0.67 (0.64, 0.70)
		Midvaal	715	0	0.00 (0.0, 0.51)
	West Rand	Kungwini	142,919	197	0.14 (0.12, 0.16)
		Merafong	71,753	275	0.38 (0.34, 0.43)
		Mogale City	238,808	2,495	1.04 (1.00, 1.09)
	*Metwseding	Nokeng Tsa Taemane	525,115	6,479	1.23 (1.20, 1.26)

*The Metsweding District together with Nokeng Tsa Taemane local municipality were closed and merged into the Tshwane Metropolitan Municipality in 2011.

^**†**^CI: Confidence interval.

**Table 4:** Characteristics of abattoirs and animal sources in Gauteng Province (South Africa) between 2010 and 2013.

Variable	Number positive for bovine cysticercosis	Number carcasses inspected	Number of bovine cysticercosis positive carcasses	Percentage (95% CI*) of Bovine cysticercosis positive carcasses
**Class of the abattoir**				
Low Throughput	34	68,217	34	0.05 (0.03, 0.07)
High Throughput	9,886	1,356,708	9,886	0.73 (0.71, 0.74)
** ** **Source of animals**				
Non-feedlot	9,920	1,373,229	9,920	0.72 (0.71, 0.74)
Feedlot	5,704	497,412	5,704	1.15 (1.12, 1.18)
** ** **Meat inspection service provider**				
Independent service provider	9,554	1,292,654	9,554	0.74 (0.72, 0.75)
Service provided by owner	366	122,351	366	0.30 (0.27, 0.33)

*CI: Confidence interval

### Determinants of bovine cysticercosis identification rates based on multivariable GEE model

Although the municipality in which the abattoir was located and the provider of the inspection services had significant simple/univariable associations with identification rates of bovine cysticercosis, the observed associations were not statistically significant in the multivariable GEE model. In addition, the number of meat inspectors in the abattoir were also not significantly associated with bovine cysticercosis identification rates.

Bovine cysticercosis identification rates of high throughput abattoirs were 9 times higher (RR: 9.4; 95% CI: 4.7–19.1) than those of low throughput abattoirs (Table 5). Similarly, the rate of identification of bovine cysticercosis was 1.6 times higher (RR: 1.6; 95% CI: 1.7–3.5) for carcasses of animals from feedlots compared to those from non-feedlot sources (Table 5).

**Table 5:** Results of the GEE Poisson model showing identified predictors/determinants of the identification rates of bovine *T*. *saginata* cysticercus in Gauteng Province (South Africa), 2010–2013.

Predictor	Relative risk	Standard error	P-value	95% Confidence interval
Class of the abattoir				
High Throughput	9.436	3.387	0.0001	4.670, 19.067
Low Throughput	Referent			
Source of Animals				
Feedlot	2.431	0.464	0.001	1.672, 3.533
Non Feedlot	Referent			

## Discussion

This study was designed to estimate cysticercosis identification rates in Gauteng Province of South Africa, and to identify predictors/determinants of variations in the identification rates. This is the first study in South Africa that investigated determinants of cysticercosis identification rates using a multivariable modelling approach. The overall cysticercosis identification rates was generally low (0.7%), although higher than the 0.2% reported in an earlier study done in the North West Province of South Africa in 2011 [18]. However, it was lower than the 1.6% reported in Matabeleland Province of Zimbabwe [13]. Due to the low sensitivity of meat inspection, as has been reported in other studies [8, 9, 20, 21], it is possible that results reported here are an underestimation of the true proportion of the cysticercosis positive carcasses. Therefore, additional studies are needed to further investigate this issue.

The significantly lower proportions of cases of bovine cysticercosis observed during the summer months (November to March) was in contrast to reports by Sungirai et al (2014) and Dzoma et al (2011) who did not observe seasonal differences in the occurrence of bovine cysticercosis cases in Zimbabwe and North West Province of South Africa respectively [13, 18]. The reason for the lower identification rates observed in the summer is unclear and will require further investigations.

The reason for the relatively high identification rates of bovine cysticercus positive carcasses in Nokeng Tsa Taemane and Mogale City is unclear. However, it could be attributed to differences in data capturing practices across abattoirs (some abattoirs might be doing a better job of recording than others) as well as differences in the rigor of meat inspection procedures and abattoir management (some abattoirs might have more experienced inspectors who do a better job of identifying the cysts). Similar reports were made by Terefe et al (2014) who observed that management practices within the abattoir affects the proportion of bovine cysticercosis positive carcasses identified and reported [22]. Moreover, other authors have reported that the accuracy of meat inspection data is dependent not only on the number of cysts present but also on the skill, rigor and number of meat inspectors employed in the abattoir [23].

The Meat Safety Act 40 of the year 2000 and associated regulations require abattoir owners to hire independent meat inspection services for their abattoirs and therefore the owners are responsible for paying for the services directly to the providers [14]. Although this arrangement seems to have a potential to lead to conflict of interest, this study found that the type of service provider was not a significant determinant of bovine cysticercosis identification rates. However, these results should be interpreted with caution, and more detailed primary base studies need to be done to specifically further investigate the role of inspection service providers in the identification and reporting of bovine cysticercosis.

Contrary to the findings of a study by Sungirai et al (2014), who reported a significantly higher prevalence of bovine cysticercosis in cattle from communal farmers compared to feedlots [13], this study found significantly higher identification rates in carcasses of cattle from feedlots as compared to those sourced from non-feedlot sources. The findings of the current study is consistent with those of other studies that have reported that animals raised on feedlots farms are more likely to be exposed to point source contamination such as sewage, feed and water [24, 25]. This has been attributed to large numbers of animals becoming infected at the same time. For example, in the USA, bovine *T*. *saginata* cysticercus outbreaks have been reported in feedlot cattle [26–28]. In Alberta, contaminated water with human sewage waste [25] and in South Wales, Australia, imported copra meal, which was used as a feed supplement have been implicated in outbreaks of *T*. *saginata* cysticercus infection among beef cattle [24].

The higher identification rates in high throughput abattoirs as compared to low throughput abattoirs contradicted the findings by Dzoma (2011), who reported a high proportion of cysticercosis in low throughput abattoirs [18]. This could be due to the fact that meat inspectors working in high throughput abattoirs are more likely to be experienced due to the large numbers of carcasses they handle resulting in the higher identification rates observed in the current study. Moreover, high throughput abattoirs have more resources to employ potentially more experienced meat inspectors.

This study used retrospective administrative data and, therefore, the investigators had no control over the quality of the data collection and hence the findings reported here should be interpreted with caution. This is because as reported by Dorny et al (2000), it is difficult to accurately estimate the proportion of cysticercosis positive carcasses based wholly on abattoir data [1]. Moreover, the data used for this study did not include some variables such as age and sex of the animal that have been reported to be associated with the occurrence of bovine cysticercosis [16, 29]. Also lacking in the records was indication of whether the cysts were alive or calcified making it difficult to directly infer risk to the consumer. Furthermore, the records did not include data on addresses of farms of origin of slaughtered animals, making it difficult to trace back the animals to the source. In view of this, there is a need to improve surveillance data collection to include these additional variables. Nonetheless, the findings of this study provide useful information on variations of cysticercosis identification rates and their predictors/determinants to guide future studies, surveillance and control efforts.

## Conclusions

Bovine cysticercosis is frequently observed in bovine carcasses in abattoirs across the Gauteng Province of South Africa, albeit at low levels. The class of the abattoir and the source of the animals were significant predictors/determinants of bovine cysticercosis identification rates. Interestingly, the number of meat inspectors and the type of service providers were not significant determinants. To better understand the differences in identification rates observed for the different variables, the authors recommend that capture of data on bovine *T*. *saginata* cysticercus infections in abattoirs need to be improved to include more variables such as the address of the farm of origin of the animal, and for each case to be reported separately to help with traceability. We further recommend that efforts to identify whether cysts are alive or calcified are needed to help better estimate risk to consumers. This study provides useful baseline data to guide future studies, surveillance data collection and control efforts.

## Abbreviations

AIC: Akaike Information Criterion
CI: Confidence interval
GDARD: Gauteng Department of Agriculture and Rural Department
GEE: Generalized Estimating Equations
RR: Relative Risk
QIC: Quasi-likelihood Independence Model Criterion
